# Comparative genomics of *Clavibacter michiganensis* subspecies, pathogens of important agricultural crops

**DOI:** 10.1371/journal.pone.0172295

**Published:** 2017-03-20

**Authors:** James T. Tambong

**Affiliations:** Ottawa Research and Development Centre (ORDC), Agriculture and Agri-Food Canada, Ottawa, Ontario, Canada; National Renewable Energy Laboratory, UNITED STATES

## Abstract

Subspecies of *Clavibacter michiganensis* are important phytobacterial pathogens causing devastating diseases in several agricultural crops. The genome organizations of these pathogens are poorly understood. Here, the complete genomes of 5 subspecies (*C*. *michiganensis* subsp. *michiganensis*, Cmi; *C*. *michiganensis* subsp. *sepedonicus*, Cms; *C*. *michiganensis* subsp. *nebraskensis*, Cmn; *C*. *michiganensis* subsp. *insidiosus*, Cmi and *C*. *michiganensis* subsp. *capsici*, Cmc) were analyzed. This study assessed the taxonomic position of the subspecies based on 16S rRNA and genome-based DNA homology and concludes that there is ample evidence to elevate some of the subspecies to species-level. Comparative genomics analysis indicated distinct genomic features evident on the DNA structural atlases and annotation features. Based on orthologous gene analysis, about 2300 CDSs are shared across all the subspecies; and Cms showed the highest number of subspecies-specific CDS, most of which are mobile elements suggesting that Cms could be more prone to translocation of foreign genes. Cms and Cmi had the highest number of pseudogenes, an indication of potential degenerating genomes. The stress response factors that may be involved in cold/heat shock, detoxification, oxidative stress, osmoregulation, and carbon utilization are outlined. For example, the *wco*-cluster encoding for extracellular polysaccharide II is highly conserved while the sucrose-6-phosphate hydrolase that catalyzes the hydrolysis of sucrose-6-phosphate yielding glucose-6-phosphate and fructose is highly divergent. A unique second form of the enzyme is only present in Cmn NCPPB 2581. Also, twenty-eight plasmid-borne CDSs in the other subspecies were found to have homologues in the chromosomal genome of Cmn which is known not to carry plasmids. These CDSs include pathogenesis-related factors such as Endocellulases E1 and Beta-glucosidase. The results presented here provide an insight of the functional organization of the genomes of five core *C*. *michiganensis* subspecies, enabling a better understanding of these phytobacteria.

## Introduction

Members of the species *Clavibacter michiganensis* (Smith 1910) are gram-positive bacteria belonging to the family Microbacteriaceae, and consist of five core subspecies. The cells are (i) rods of coryneform morphology, (ii) having B2γ-type cell wall peptidoglycan with the diaminobutyric acid MK-9 as the predominant menaquinone, (iii) phosphatidyglycerol and diphosphatidyglycerol as the basic polar lipids, and (iv) a high GC content of 72–74 mol% [1,2]. All of the subspecies are plant pathogens of important agricultural crops (attacking members of the *Solanaceae*, *Poaceae*, and *Leguminosae*). Given the high level economic threat that they can cause, four of these subspecies are categorized as quarantine phytosanitary organisms [3]. They cause diseases of tomato (*C*. *michiganensis* subsp. *michiganensis* =, Cmm), potato (*C*. *michiganensis* subsp. *sepedonicus* =, Cms), alfalfa (*C*. *michiganensis* subsp. *insidiosus* =, Cmi), corn (*C*. *michiganensis* subsp. *nebraskensis* =, Cmn) and pepper (*C*. *michiganensis* subsp. *capsici* =, Cmc). Latent systemic infections of the xylem can be caused by all subspecies. All subspecies have been reported to invade seeds, and seems to poorly survive in soil [4,5]. In addition, they may have an epiphytic saprobic mode [6].

The genome organizations of subspecies of *Clavibacter michiganensis* are poorly understood. Next-generation technologies have revolutionized genome sequencing and as such the number of bacterial genomes available for analysis is expanding rapidly [7,8], leading to the generation of complete chromosomal and plasmid genomes of representatives strains of five subspecies (Cmm, Cms, Cmi, Cmn and Cmc) of *C*. *michiganensis*. Detailed analyses of the genomes of Cmm and Cms identified new sets of pathogenicity-related genes [9,10]. In Cmm and Cms, plasmid-borne virulence factors have been implicated in disease induction while chromosomally encoded genes are involved in successful host colonization [11]. In Cmm, a 129-kb low G+C region ((*chp*/*tom*A) near the origin of replication was considered essential for pathogenicity [10]. For example, individual genes found in this region, such as serine proteases, are necessary for effective colonization of tomato [10]. The serine protease-encoding *pat*-1 gene and cellulase-encoding *cel*A gene in Cmm are directly implicated in pathogenicity [12]. An intact orthologue occurs in Cms. However, *cel*B, a second cellulase gene, on the genome of both subspecies, is deactivated by a nonsense mutation in Cms [9]. It is unclear whether similar or novel regions exist in the genomes of Cmn, Cmi and Cmc. The complete chromosomal genome sequences of Cmn strain NCPPB 2581 (K.H. Gartemann, GenBank accession # HE614873), Cmi strain R1-1 [13] and Cmc strain PF008 [14] were published. The Cmi genome carries 3 plasmids while that of Cmc has two plasmids which might possess similar virulence factors. The genome of Cmn 2581 is not known to carry plasmids (Gartemann, per. Comm). Plasmids are reported not to be required for the pathogenicity of Cmn since most strains isolated do not carry a plasmid [11,15]. As such, it is suggested that the virulence mechanisms might be different from those reported for Cmm or Cms [16]. Since the genome of Cmn 2581 does not carry any plasmids, it can be hypothesized that the disease-inducing virulence factors are also chromosomally encoded alongside genes involved in successful host colonization. However, *Clavibacter michiganensis* subspecies harboring plasmid-borne disease-inducing virulence factors on the chromosome is yet to be reported.

The goals of this study were (i) to assess the taxonomic position of the subspecies based on 16S rRNA and genome-based DNA-DNAhomology; ii) to perform a comprehensive comparison of genomes of Cmm, Cms, Cmc, Cmi and Cmn using DNA structural and annotation features; (iii) to identify some of the genes involved in survival capacity and carbon utilization; and (iv) to assess whether some of the disease-inducing plasmid-borne virulence factors are present on the chromosomal genome of Cmn strain NCPPB 2581. Analyses of DNA structural features of complete genomes can pinpoint genomic regions that are sites of certain genes and elements involved in significant biological processes. Analyzing genome sequences can confer a wide range of new knowledge [17,18] useful in highlighting species and subspecies diversity that would not be otherwise possible [19].These will enable a better understanding of the host-specificity and pathogenicity of the subspecies of *C*. *michiganensis* and identify evolutionary genomic events associated with subspeciation [9]. The results presented here suggest that most of the subspecies could be distinct species. Comparative genomics revealed that the *wco*-cluster involved in extracellular polysaccharide II production is conserved within the subspecies while the sucrose-6-phosphate hydrolase is not; and outlined genes that may be implicated in stress responses. Finally, the data also show that some plasmid-borne genes in Cmm, Cms, Cmi and Cmc are chromosomally encoded in Cmn, known to not carry plasmids.

## Materials and methods

### Genome downloads and annotation

Whole-genome data of the five *C*. *michiganensis* subspecies were downloaded from GenBank [20] at NCBI, www.ncbi.nlm.nih.gov/genome/browser. NCBI GenBank International Nucleotide Sequence Database Collaboration (INSDC) or Whole-genome-sequence (WGS) numbers was used, respectively, to download each genome in the NCBI GenBank format using the getgbk.pl script as implemented in CMG-Biotools [19]. Genome sequences were extracted from GenBank files and saved in FASTA format using the saco_convert script [21]. The complete genomes of the 5 subspecies were submitted to the RAST web-based annotation system [22] and PATRIC [23] followed by manual curation.

### Basic characterization of genomes

16S rRNA and *gyr*B-*rec*A-*rpo*B phylogenies of *Clavibacter michiganensis* subspecies were implemented in MEGA7 [24] using neighbor-joining method with Kimura 2-parameter and Jukes-Cantor models respectively. Branch robustness was evaluated using 1000 bootstrap replicates. genome-sequence-based digital DNA-DNA hybridization (dDDH; [25]) and MUMmer-based average nucleotide identity (ANIm;[26]) were employed to assess the taxonomic position of strains relative to the closest taxon, *Rathayibacter tritici* NCPPB 1953 (GenBank # CP015515). The dDDH values were calculated using the genome-to-genome distance calculator (GGDC) Version 2.1 (http://ggdc.dsmz.de; [25]). ANIm similarity values were computed as described by Kurtz et al. [26] and implemented in JSpecies [27].

### Genome comparison and analysis

The structural DNA atlases were generated from complete genomes as implemented in CMG-Biotools [19,28] to show the average and standard deviation of percent AT, GC skew, global repeats, intrinsic curvature and stacking energy. Each of the parameters are computed independently through a pipeline and outputted in a circular plot, an atlas [17].

#### Proteome comparisons

The comparison of proteomes was implemented using PATRIC web service [23]and CMG-Biotool [19]. PATRIC was executed using default parameters. For CMG-Biotool, a blastmatrix was generated using an XML formatted input file created by makebmdest [19]. A pairwise proteome comparison using BLAST [29] was used to generate a BLAST matrix. Protein sequences were compared to each other. Two sequences are similar and collected in the same ‘‘protein family’ if the BLAST hit had at least 50% identical matches in the alignment and the length of the alignment is 50% of the longest gene in the comparison. For the comparison of two genomes, single linkage is used to build protein families. Paralogs within a proteome are also evaluated and outputted at the bottom row of the matrix. Also, the Protein Family Sorter tool of PATRIC [23] was used to examine the distribution of specific gene families, known as FIGFams, across the different genomes. Analysis of orthologous clusters was also performed using the FastOrtho (http://enews.patricbrc.org/fastortho/), a faster reimplementation of OrthoMCL [30] with default parameters (e-vlaue of 1e^-5^ and inflation value of 1.5).

## Results

### Verification of strain identity and genomic relationship

Since Cmi strain R1-1 and Cmm strain NCPPB 382 were not type strains, their identities were verified. The 16S rDNA extracted of Cmi R1-1 and NCPPB 382 genomes were compared to those of their corresponding type strains by BLAST and phylogenetic analysis. Nucleotide BLAST searches (http://blast.ncbi.nlm.nih.gov/Blast.cgi) of the GenBank database showed that the 16S rDNA sequences of both strains exhibited more than 99% nucleotide identities to their respective type strains, LMG 3663^T^ (U09761) and DSM 46364^T^ (X77435). Seventeen 16S rDNA sequences from different subspecies and closely related genera (*Rathayibacter* and *Leifsonia*) were selected to infer a phylogenetic tree that showed strains Cmi R1-1 and Cmm NCPPB 382 clustered perfectly with their respective type strains (S1 Fig).

**Fig 1:**
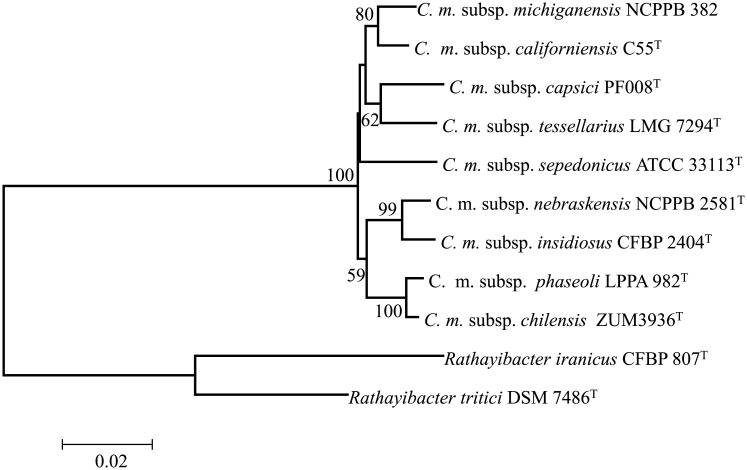
*gyr*B-*rec*A-*rpo*B phylogeny of core *Clavibacter michiganensis* subspecies inferred using the neighbor-joining method and conducted in MEGA7[24]. The optimal tree with the sum of branch length = 0.31515422 is shown. The evolutionary distances were computed using the Jukes-Cantor method. The percentage of replicate trees in which the associated taxa clustered together in the bootstrap test (1000 replicates) are shown next to the branches. Bootstrap values less than 50 are not shown.

Genome similarity analysis using dDDH and ANIm showed values ranging from 39.1 to 60% and 90.75–95.25% respectively (Table 1). All the dDDH values are below (70%) the proposed cut-off species boundary. Highest dDDH homology (60%) was between Cmi and Cmn and the lowest was was Cms and Cmc. Similar trend was observed for ANIm values (cut-off = 95%) with the exception of Cmi-Cmn value that was 95.2% (Table 1). A well-supported *gyr*B-*rec*A-*rpo*B phylogeny (Fig 1) of *C*. *michiganensis* subspecies is in agreement with dDDH and ANIm results.

**Table 1:** Chromosomal genome similarities between *Clavibacter michiganensis* subspecies based on genome-to-genome digital DNA-DNA Hybridization (dDDH; lower diagonal) and MuMmer-based Average Nucleotide Identity (ANIm; upper diagonal).

**Subspecies**	Cmm	Cms	Cmi	Cmn	Cmc
Cmm	100	92.48%	93.11%	92.88%	91.05%
Cms	46.3% [43.7–48.9]	100	92.32%	92.34%	90.79%
Cmi	48.7% [46.1–51.3]	45.20% [42.6–47.7]	100	95.25%	91.11%
Cmn	48.01% [45.4–50.6]	45.2% [42.6–47.8]	60.00% [57.2–62.8]	100	91.18%
Cmc	40.2% [37.7–42.7]	39.1% [36.6–41.6]	40.5% [38.0–43.0]	40.7% [38.2–43.3]	100

dDDH values were computed using the program GGDC 2.1 [25], the model confidence intervals are shown in square brackets. ANIm values were computed in Jspecies program [27]; Cmm, *Clavibacter michiganensis* subsp. *michiganensis*; Cms, *C*. *michiganensis* subsp. *sepedonicus*; Cmi, *C*. *michiganensis* subsp. *insidiosus;* Cmn, *C*. *michiganensis* subsp. *nebraskensis;* Cms, *C*. *michiganensis subsp*. *capsici*.

### Summary statistics and general features of the genomes

The basic statistics and general features of the five *C*. *michiganensis* subspecies genomes are shown in Table 2. The genome sizes ranged from 3.06 (Cmn) to 3.41 Mb (Cmi). All the subspecies genomes possess 2 or 3 plasmids except Cmn that has no plasmid. High G+C content (72.42–73.19%) is characteristic of actinomycetes. The number of protein-coding genes with function is between 2201 (Cmn) and 2341 (Cmi) with 18 to 114 pseudogenes (Table 2). The atlases of these *C*. *michiganensis* subspecies visually represent structural properties of the genomic DNA molecule such as intrinsic curvature, stacking energy, position preference, and inverted and direct repeats. S1 Fig. shows DNA structures of the five genomes including the locations of rRNA operons, inverted and direct repeats as well as strongly curved regions (high stacking energy and DNA intrinsic curvature) having genes that might be involved in a functionally specific DNA structure. The genome atlas of Cms ATCC 33113 exhibits, at least, 31 inverted repeat regions while Cmi R1-1 has about 14 (S2 Fig). The other subspecies lacked any visible inverted repeats. All the subspecies have three or more direct repeats, with Cms ATCC 33113 having the highest number of direct repeats (S2 Fig).

**Table 2:** General genome features of the five core *Clavibacter michiganensis* subspecies.

Genome feature	*Clavibacter michiganensis* subspecies				
	Cmm	Cms	Cmi	Cmn	Cmc
Size (bp)	3,395,237	3,403,786	3,408,062	3,063,596	3,241,713
No. of plasmids	2	2	3	0	2
G + C content	72.53	72.42	72.64	73.00	73.19
Protein-coding genes^a^	3105	3245	3386	2909	3144
No. of protein-coding genes with function	2319 (74.7%)	2263 (69.7%)	2341 (69.1%)	2201 (75.6%)	2269 (72.2%)
No. of protein-coding genes without function-hypothetical	786 (25.3%)	982 (30.3%	1045 (30.9%)	708(24.4%)	875 (27.8%)
No. of protein-coding genes with EC number assignments	769	726	784	764	763
No. of protein-coding genes with GO assignments	773	734	693	676	674
No. of protein-coding genes with pathway assignments	607	571	616	605	600
tRNA genes	45		46	45	45
rRNA genes (5S, 16S, 23S)^b^	(2, 2, 2)	(2, 2, 2)	(2, 2, 2)	(2, 2, 2)	(2, 2, 2)
ncRNA genes^b^	1		1	1	1
Pseudogenes^b^	18	114	109	50	79
Conserved CDS^c^	2300	2301	2301	2295	2301
Subspecies-specific CDS^d^	12	125	39	6	28

^a^ chromosomal and plasmid protein-coding genes where applicable;

^b^ data from GenBank files;

^c^ protein-coding genes having orthologous genes in every subject genomes;

^d^ protein-coding genes not having orthologous genes in any other subject genome.

The orthologous relationship was computed using FastOrtho. GO, Gene ontology. EC number, Enzyme Commission number.

### Comparative genomics

**Fig 2:**
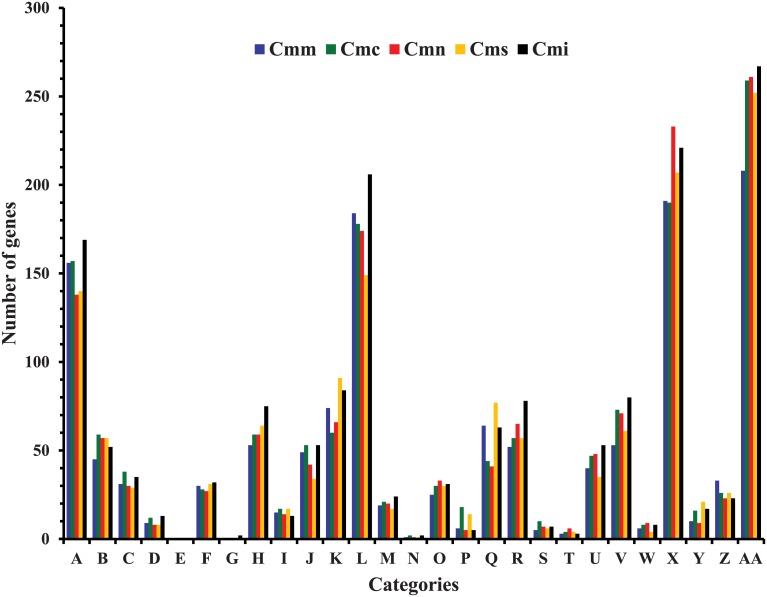
Comparison of functional categories among five subspecies of *Clavibacter michiganensis*. The ordinate axis resents the number of genes in each functional category. The 27 categories are: Cofactors, vitamins, prosthetic groups, pigments **(A**); Cell wall and Capsule (**B**); Virulence, disease and defense (**C**); Potassium metabolism (D); Photosynthesis (**E**); Miscellaneous (**F**); Phages, prophages, transposable elements, plasmids (**G**); Membrane transport (**H**); Iron acquisition and metabolism (**I**); RNA metabolism (**J**); Nucleosides and nucleotides (**K**); Protein metabolism (**L**); Cell division and cell cycle (**M**); Motility and chemotaxis (**N**); Regulation and cell signaling (**O**); Secondary metabolism (**P**); DNA metabolism (**Q**); Fatty acids, lipids, and isoprenoids (**R**); Nitrogen metabolism (**S**); Dormancy and sporulation (**T**); Respiration (**U**); Stress response (**V**); Metabolism of aromatic compounds (**W**); Amino acids and derivatives (**X**); Sulfur metabolism (**Y**); Phosporus metabolism (**Z**); Carbohydrates (**AA**). Cmm, *Clavibacter michiganensis* subsp. *michiganensis;* Cmc, *C*. *michiganensis subsp*. *capsici;* Cmn, *C*. *m*. *subsp*. *nebraskensis;* Cms, *C*. *m*. *subsp*. *sepedonicus;* Cmi, *C*. *m*. *subsp*. *insidiosus*.

**Fig 3:**
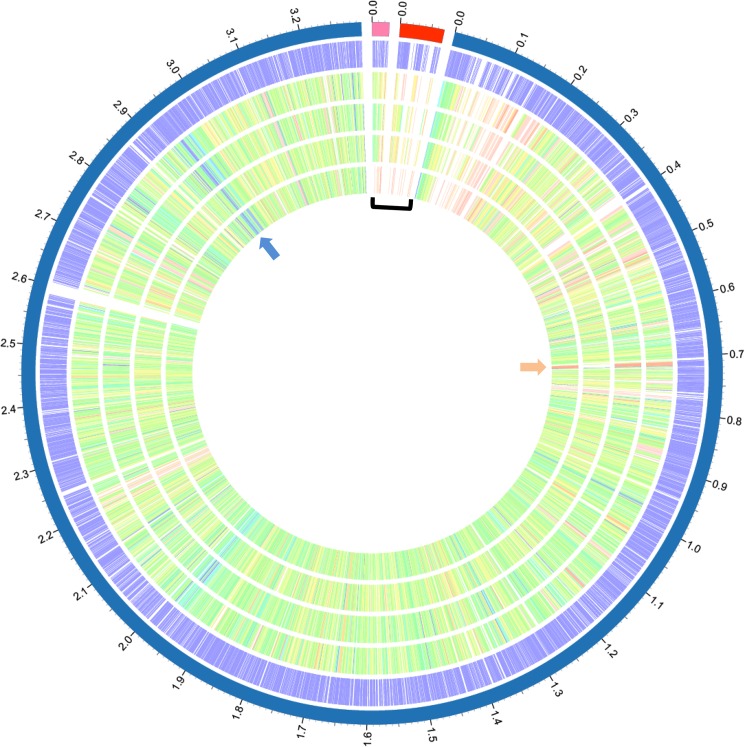
Proteomes comparison of the genomes of the five *Clavibacter michiganensis* subspecies. The outermost circle (circle 1) represents the scale (Mb) of the chromosomal (blue) and plasmids (orange and red) of *C*. *michiganensis* subsp. *mich*iganensis NCPPB 382. Circle 2, the chromosomal and plasmids protein sequences of *C*. *m*. subsp. *michiganensis* NCPPB 382 as references; circle 3, protein sequences of *C*. *michiganensis* subsp. *sepedonicus* ATCC33113; circle 4, protein sequences of *C*. *michiganensis* subsp. *insidiosus* R1-1; circle 5, protein sequences of *C*. *m*. subsp. *capsici* PF008; circle 6, protein sequences of *C*. *m*. subsp. *nebraskensis* NCPPB 2581. Protein sequences are represented by colorful sticks blue(100%)-to-brown (10%) were assigned according to the protein homolog in NCPPB 382 genome) in circles 2 to 6. Blue arrow depicts conserved protein family (e.g. LSU ribosomal protein, L14p); brown arrow, species-specific protein family (e.g. putative large secreted protein); and square bracket protein families in plasmids with homologues in chromosomal genome of Cmn NCPPB 2581.

**Fig 4:**
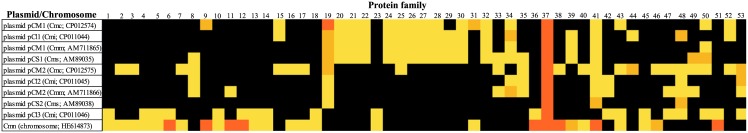
The chromosome of *Clavibacter michiganensis* subsp. *nebraskensis* NCPPB 2581 (Cmn) contains protein families with homologies in at least one of the plasmids of the other subspecies (Cmm, Cms, Cmc and Cmi). **1**, **Transaldolase (EC 2.2.1.2)**; **2**, **Na+/H+ antiporter NhaA type**; **3**, **Beta-glucosidase (EC 3.2.1.21**); **4**, **Inosose dehydratase (EC 4.2.1.44**); **5**, **5-deoxy-glucuronate isomerase (EC 5.3.1.-); 6, Beta-hexosaminidase (EC 3.2.1.52); 7, Alpha-galactosidase (EC 3.2.1.22); 8**, Chromosome (plasmid) partitioning protein ParB; **9**, **Transcriptional regulator, ArsR family**; **10**, **5-keto-2-deoxy-D-gluconate-6 phosphate aldolase [form 2] (EC 4.1.2.29); 11**, **putative esterase**; **12**, **Long-chain-fatty-acid—CoA ligase (EC 6.2.1.3**); **13**, **5-keto-2-deoxygluconokinase (EC 2.7.1.92)**; **14**, **Epi-inositol hydrolase (EC 3.7.1.-)**; **15**, ATP-binding protein p271; **16**, **Possible alpha-xyloside, ABC transporter, permease component**; **17**, **Possible alpha-xyloside, ABC transporter, substrate-binding component**; **18**, **N-Acetyl-D-glucosamine ABC transport system, permease protein 2**; **19**, FIG00511136: hypothetical protein; **20**, FIG00511175: hypothetical protein; **21**, FIG00511336: hypothetical protein; **22**, FIG00511343: hypothetical protein; **23**, **FIG00511395: hypothetical protein**; **24**, FIG00511567: hypothetical protein; **25**, FIG00511653: hypothetical protein; **26**, FIG00512013: hypothetical protein; **27**, FIG00512097: hypothetical protein; **28**, FIG00512209: hypothetical protein; **29**, hypothetical protein, putative partitioning protein; **30**, hypothetical protein, putative transcriptional regulator ArsR family; **31**, **putative cation efflux protein, CDF family**; **32**, Protein containing ATP/GTP-binding site motif A; **33**, FIG00512364: hypothetical protein; **34**, FIG00512599: hypothetical protein; **35**, Single-strand binding protein homolog Ssb; **36**, **Transcriptional regulator, GntR family**; **37**, **hypothetical protein**; **38**, **Transcriptional regulator, LacI family**; **39**, **Endoglucanase E1 precursor (EC 3.2.1.4) (Endo-1,4-beta-glucanase E1) (Endocellulase E1)**; **40**, Membrane protein mosC; **41**, putative secreted protein; **42**, elements of external origin; phage-related functions and prophages; **43**, **Secreted protein**; **44**, V8-like Glu-specific endopeptidase; **45**, **Methylmalonate-semialdehyde dehydrogenase [inositol] (EC 1.2.1.27)**; **46**, **Rhodanese-related sulfurtransferase**; **47**, Chromosome (plasmid) partitioning protein ParA; **48**, Mobile element protein; **49**, Cell filamentation protein; **50**, DNA invertase; **51**, **Myo-inositol 2-dehydrogenase (EC 1.1.1.18)**; **52**, Tn552 transposase; **53**, lysyl tRNA synthetase-like protein. Protein families in bold indicate families identified on the Cmn chromosome and at least one plasmid. Cmm, *Clavibacter michiganensis* subsp. *michiganensis*; Cms, *C*. *m*. subsp. *sepedonicus*; Cmc, *C*. *m*. subsp. *capsic*i; Cmi, *C*. *m*. subsp. *insidiosus*. Information was generated using Protein Family Sorter module (FIGfams) of PATRIC [23]. Black, no corresponding protein family; yellow, one protein-coding sequences (CDS) present; Golden yellow, two CDS present; and orange, three or more CDS present.

Comparison of the functional categories among the five subspecies of *C*. *michiganensis* shows that the highest number of CDSs that are involved in carbohydrate metabolism, while none is involved in photosynthesis (Fig 2). Pairwise proteome comparisons using the BLAST matrix [19] between the genomes showed similarity ranging from 66.1% to 74.6% (S3 Fig). The genome of Cmm NCPPB 382 has the highest similarity (75.3%) to that of Cmn NCPPB 2581 (S3 Fig). Cms exhibited the lowest proteome similarities (66.4–68.8%) with the other *C*. *michiganensis* subspecies (S3 Fig). To identify conserved and subspecies-specific CDSs, pan-genome analyses including orthologous group classification and orthologous relationship were performed. Orthologous relationships were determined using the FastOrtho method. All the CDSs of the five subspecies were clustered into 3,155 orthologous groups with 2,274 conserved groups. The number of conserved protein-coding sequences is relatively similar across the different subspecies (Table 2). Cmn NCPPB 2581 has the lowest number of subspecies-specific CDSs while Cms has the highest number (Table 2). PATRIC proteome comparison tool was used to compare the genomes of the five *C*. *michiganensis* subspecies. An overview of the conserved (blue arrow) and specific (brown arrow) genomic regions are given in Fig 3. Also, some of the plasmid-borne CDSs showed homologies to chromosomal genome of Cmn NCPPB 2581 known to not carry plasmids (Fig 3; square bracket). Twenty-eight plasmid-borne CDSs are present in the chromosomal genome of Cmn which include pathogenesis-related factors such as Endocellulases E1 and Beta-glucosidase (Fig 4). At least 75 CDSs related to stress response were identified in the genomes after analysis and comparison (S1 Table). These include CDSs involved in oxidative and osmotic stresses, cold and heat shock, and resistance to antibiotics and toxic compounds.

## Discussion

This study compared, for the first time, the complete genomes of five *C*. *michiganensis* subspecies. Of the 5 strains analysed, two (R1-1 and NCPPB 382) are not type strains. However, based on 16S rDNA BLAST and phylogenetic these strains were confirmed to belong to the same taxonomic positions as their corresponding type strains. Genome comparisons of the subspecies based on dDDH and ANIm showed values that are significantly below the cut-off threshold for species delineation, suggesting a higher taxonomic position (species-level) for these bacteria. A formal taxonomic study will provide a better insight.

Comparative genomic analysis of genomes showed that Cmn has the smallest genome, resulting in the fewest number of protein-coding genes, suggesting that colonizing and living in corn leaf tissues requires relatively few genes. Proteome comparison revealed that the Cms has the lowest similarity to the other *C*. *michiganensis* subspecies, suggesting that Cms is a more divergent probably linked to its soil niche, a more complex environment. Also, the Cms ATCC 33113 genome showed highest number of direct repeats most of which are mobile elements constituting most of the subspecies-specific protein-coding genes. Direct repeats play a significant role in the diversification of *Helicobacter pylori* DNA [31,32]. Wide-ranging repetitive DNA could facilitate the plasticity of a prokaryotic genome [31], suggesting that the genome of Cms ATCC 33113 could be more prone to translocation of foreign genes than the other subspecies. Also, the genomes of Cms and Cmi had the highest number of non-functional pseudogenes which might reduce the coding capacity of these strains, suggesting possible degeneration of the genome [9]. This process is often associated with new niche adaptation by a bacterial species, making certain gene expendables [9,10].

The genomic DNA atlases also revealed differences in intrinsic curvatures. High curvature and stacking energy regions, for example, in Cmm NCPPB 382 (S1 Fig; brown arrows) indicate strongly curved regions that might be involved in specific biological function. Curved DNA portions seems to have highly expressed genes that are modulated by histone-like proteins [19]. The rRNA operons are associated with regions of high curvature, average stacking energy and low position preference in all the chromosomal genomes of the subspecies. DNA curvature plays a significant role in several biologically vital processes, including recombination [33], DNA replication [34], and positioning of nucleosome [35].

Comparisons of functional categories among the genomes of the five subspecies showed that the number genes implicated in carbohydrate metabolism and transport (Fig 2, category AA) were highest compared to the other categories within each genome. This suggests that carbohydrate metabolism is a key factor to the survival of these subspecies and could be involved in plant-pathogenic interaction. For example, *in planta*, genes within the *wco*-cluster involved in sugar metabolism were up-regulated in Cmm in late infection stages suggesting potential involvement in pathogenicity [36]. The functions of genes in this cluster include chitinases, putative glycosyltransferase, glycoamylases and GumJ proteins. In a tomato plant study [36] in Cmm, the CMM_0824 locus encoding for glycosyltransferase (*wco*F) showed highest up-regulated value. Also, the GumJ protein contributes to the formation of biofilm and cells adhesion to host surfaces [37–40]. Genome-wide comparison of the five subspecies showed that the genes within this cluster are generally conserved (90–99%). Seventeen CDSs were identified in all the genomes. Three CDSs identified on the Cmm genome as *wco*A, *wco*B and *wco*P had low homology in the other genomes. *wco*A, a chitinase, in Cmm had only 67.3% similarity to a potential homologous gene in Cmc. A hypothetical protein (*wco*B) in Cmm had low similarities to CDSs in Cms (85%), Cmc (66.4%) and Cmn (68.7%). A transcriptional regulator of the MarR family (*wco*P) present in Cmm showed about 86.8% and 36.6% in Cmn and Cmc, respectively. Given their up-regulation *in planta*, these genes may play an important role in utilizing plant derived nutrients.

Sucrose is a naturally abundant carbohydrate found in several plants and plant parts (Reid and Abratt, 2005). A CDS encoding for sucrose phosphate synthases associated with sucrose biosynthesis was identified in all the subspecies and showed about 93.0% homology to locus CMM_0494 found in Cmm. Two CDSs associated with sucrose catabolism were identified but only one is present in all the subspecies. Sucrose phosphorylase (S1–S5 Datasets), an important enzyme that converts sucrose to D-fructose and alpha-D-glucose-1-phosphate, is present in all the subspecies with about 87.8% homology to locus CMM_2523 found in Cmm. However, a sucrose-6-phosphate hydrolase (EC 3.2.1.26) found in Cmm (CMM_2780) was identified only in Cmn and Cms (CMS_0938) with a low homology of 36.4% and 49.8% respectively. A second CDS encoding another form of sucrose-6-phosphate hydrolase (EC 3.2.1.B3) is present only in Cmn, the pathogen of corn. Corn possesses a very active sucrose-6-phosphate biosynthetic system. Cytoplasmic sucrose-6-phosphate hydrolase catalyzes the hydrolysis of sucrose-6-phosphate yielding glucose-6-phosphate and fructose [41]. It is unclear why this high divergence among the subspecies especially its absence in Cmi and Cmc. It is possible that alternate pathways exit in Cmi and Cmc. In *Streptococcus mutans*, Tao et al. [42] indicated that other sugar transport including sucrose is done through the MSM (multiple sugar metabolism) systems.

The survival of bacteria in a given environment depends on the ability to respond to changes in oxidative stress. At least 22 CDSs involved in oxidative stress response were identified in all the subspecies (S1 Table). The CDSs coding for catalases (EC 1.11.1.6), superoxide dismutases (EC 1.15.1.1), and ferroxidases (EC 1.16.3.1) are conserved among the five genomes of the subspecies with homology of about 99%. Other CDSs found in all the subspecies include iron-binding ferritin-like antioxidant protein and alkyl hydroperoxidase reductase subunit C-like protein. In addition, all the *Clavibacter* subspecies genomes encode glutathione peroxidase (EC 1.11.1.9). Also, a CDS encoding for redox-sensitive activator (SoxR), an oxidative stress response protein; *fur*B, a zinc uptake regulation protein (ZUR), and a transcriptional regulator of the FUR family are present in all the genomes studied.

All *Clavibacter* subspecies encode 5 CDSs involved in biosynthesis of mycothiol, an unusual thiol compound found in the Actinobacteria with important antioxidant and detoxification functions[43]. A CDS, *msh*A encodes N-acetylglucosamine transferase involved in the formation of GlcNAc-Ins; *msh*B encodes for deacetylase; *msh*C (ligase) catalyses the ligation of GlcN-Ins with a cysteine [44] followed by the acetylation of Cys-GlcN-Ins to form mycothiol. This acetylation process is catalysed by *msh*D, acetyltransferase [45]. The fifth CDS is the mycothiol S-conjungate amidase, Mca. Mca is involved in the cleavage of the amide bond of mycothiol S-conjugates of specific xenobiotics and alkylating agents producing mercapturic acid and GlcN-Ins excreted from the cell [43]. While Mca had a homology level of 96% among the subspecies, lower homology values (89.3–91.0%) were observed for genes involved in mycothiol biosynthesis.

In Actinobacteria, mycothiol biosynthesis is also implicated in arsenate resistance [46], a process that involves chemically reducing the toxic arsenate. The reduction of the product arseno-mycothiol is catalysed by mycoredoxin (EC 1.20.4.3) to mycothiol-mycoredoxin disulfide and arsenite followed by the formation of mycothione by a second mycothiol that recycles mycoredoxin. In the genomes of *Clavibacter* subspecies, CDSs linked to arsenic resistance are chromosomally and plasmid encoded except for Cmn 2581 where it is in the chromosome only. Two CDSs, *ars*B encoding arsenic efflux pump protein and *ars*C2 encoding arsenate-mycothiol transferase (EC 2.8.4.2) are present in all the chromosomes of the subspecies with high homology. Also, three CDSs encoding the arsenic transcriptional repressor (arsR) are present in the chromosome of all the genomes. In addition, one CDS of arsR is carried in the plasmids of all the subspecies except the Cmn which has no plasmid. The lack of plasmid in Cmn can suggest a low tolerance to arsenic. In the *Staphylococcus* [47,48]- or *E*. *coli* R773 or R46 [49,50]the plasmid-borne operons confer considerably high level of arsenic resistance than the chromosomal operon.

In addition to arsenic tolerance, the survival of bacteria in their respective ecological niches is dependent on their resistance to antibiotics and toxic compounds including metals such as selenium and copper. Bacteria have developed effective homeostasis and resistance systems in order to maintain the required functional amounts of these metals while detoxifying excesses. These complicated processes involve acquisition, sequestration, and efflux of metal ions [51]. Selenium occurs naturally in the Earth’s crust; and at low concentration it is essential for living organisms [52]. Under aerobic conditions, this trace element exists as selenite and selenate, and at high levels these salts can be toxic and mutagenic to bacteria [52,53]. High selenite-resistant bacterial strains like *Ralstonia metallidurans* CH34 possess the *ded*A gene that regulatesselenite uptake [52,53]. Three *ded*A genes encoding the putative selenite transport protein (DedA) including various polyols permease components of the ABC transporters are present in each of the *Clavibacter* subspecies, suggesting that members of the species *C*. *michiganensis* can detoxify environmental selenite/selenite.

In addition, Copper, an essential trace and redox-active element, serves as a cofactor for several enzymes. In aerobic cells, excess Cu metal ion can produce cytotoxic reactive oxygen species capable of damaging DNA, lipids and proteins[51]. A CDS that is chromosomally encoding Copper-translocating P-type ATPase (*cop*A; EC 3.6.3.4), repressor CsoR of the *cop*ZA operon, and Copper (I) chaperone CopZ; two CDSs each encoding for Copper resistance protein CopC and conserved membrane protein in copper uptake (YcnI) are present in all the subspecies. In addition, all the genomes have one CDS encoding for *cop*D (a Copper resistance protein) except for Cmc PF008 that has two CDSs encoding for CopD. It might be interesting to elucidate why Cmc PF008 has more than one copy of the *cop*D. Other stress response factors found in all the genomes include sigma factors (*Rsb*W, *Rsb*V, *Sig*B, *Rsb*U), Hfl operon encoding the GTP-binding protein, bacterial hemoglobin-like protein (HbO). Each subspecies has a CDS for HbO.

Cold- and heat-shock responses enable bacteria to survive changes in environmental temperature [54]. The cold shock response is governed by the expression of RNA chaperones and ribosomal factors. Two cold-shock protein (*csp*A and *csp*C) genes were identified in each of the *Clavibacter michiganensis* subspecies. In *Escherichia coli*, *csp*C, reported previously to be a regulator of *rpo*S [55], is expressed at 37°C and involved in cell division [56,57]. Bacterial responses to heat shock include heat shock proteins (HSPs) that are encoded by transcriptional up-regulation of genes. Genome of all the subspecies have a dnaK gene that encodes for heat-shock protein GrpE, chaperone proteins DnaJ and DnaK, a transcriptional repressor of the dnaK operon (*hsp*R), *hrc*A, a heat-inducible repressor of transcription, and other genes (e.g. *smp*B, encoding HSPs). With the exception of the chaperone *grp*E, cold- and heat-shock response proteins are conserved (homology of 96–99%) among the subspecies. Cmi and Cmn exhibited a 98% homology with the protein GrpE while both had only a 91% similarity to the other *C*. *michiganensis* subspecies.

This study assessed the taxonomic position of the subspecies based on 16S rRNA and genome-based DNA homology and concludes that there is ample evidence to perform a detailed analysis to elevate some of the subspecies to species-level. In addition, a detailed comparative genomics of the genomes of the subspecies indicated distinct genomic features evident on the DNA structural atlases and annotation features. Orthologous gene analysis revealed that the about 2300 CDSs are conserved across all the subspecies; and Cms showed the highest number of subspecies-specific CDS, most of which are mobile elements, suggesting that Cms could be more prone to translocation of foreign genes. In addition, Cms Cmi had the highest number of pseudogenes, an indication of potential degenerating genomes. This study also summarized some of the genetic factors encoded in these subspecies to survive under different stress conditions. The study outlined some of the stress response factors that may be involved in cold/heat shock, detoxification, oxidative stress, osmo-regulation and carbon utilization. In carbon utilization, the *wco* cluster encoding for extracellular polysaccharide II is highly conserved while the sucrose-6-phosphate hydrolase that catalyzes the hydrolysis of sucrose-6-phosphate yielding glucose-6-phosphate and fructose is highly diverged. It will be intriguing to elucidate why this gene is absent in Cmc and Cmi. The results presented here provide an insight of the functional organization of the genomes of five *C*. *michiganensis* subspecies and as such a better understanding of these phytobacteria.

## Supporting information

S1 Dataset Annotated features of *Clavibacter michiganesis* subsp. *michiganensis* strain NCPPB 382 generated by PATRIC [23]. (XLSX)

S2 Dataset Annotated features of *Clavibacter michiganesis* subsp. *sepedonicus* strain ATCC 33113 generated by PATRIC [23]. (XLSX)

S3 Dataset Annotated features of *Clavibacter michiganesis* subsp. *nebraskensis* strain NCPPB 2581 generated using PATRIC [23]. (XLSX)

S4 Dataset Annotation features of *Clavibacter michiganesis* subsp. *insidiosus* strain R1-1 generated using PATRIC [23]. (XLSX)

S5 Dataset Annotated features of *Clavibacter michiganesis* subsp. *capsici* strain PF008 generated by PATRIC [23]. (XLSX)

S1 Fig 16S rDNA phylogenetic tree of *Clavibacter michiganensis* strains inferred using the neighbor-joining method^1^ implemented in MEGA7 [24]. The optimal tree with the sum of branch length = 0.11186829 is shown. The values next to the branches are percentage of replicate trees in which the associated taxa clustered together in the bootstrap test (1000 replicates). Bootstrap values greater than 50% are shown. The tree is drawn to scale, with branch lengths in the same units as those of the evolutionary distances used to infer the phylogenetic tree. The evolutionary distances were computed using the Kimura 2-parameter method. The analysis involved 17 nucleotide sequences. All positions containing gaps and missing data were eliminated. There were a total of 1,250 positions in the final dataset. Taxa in bold are strains used in genome comparison that are not type strains and clustered perfectly with the corresponding type strains. The sequence accession numbers of the taxa are given in parentheses.
(PPTX)

S2 Fig DNA structures of complete genomes of the five *Clavibacter michiganensis* subspecies based on genomic atlases. Data of DNA, RNA and gene annotation are from the published GenBank entries. Each lane of the circular representation of the chromosome shows a different DNA feature. From innermost circle: size of genome (axis), percent AT (red = high AT), GC skew (blue = most G’s; orange arrows), inverted and direct repeats (color = repeats), position preference, stacking energy and intrinsic curvature. Dark brown arrows highlight areas of the genome with significantly different DNA structures than the remaining of the genome. Blue arrows shows the locations of rRNA operons as annotated in the GenBank file. Genome atlas was generated using CMG-Biotools [19] which calculates a numerical value for each nucleotide and saved in a file that is read by GeneWiz software. See “Materials and Methods” for details.
(PPTX)

S3 Fig BLAST matrix of proteomes of five strains of *Clavibacter michiganensis* subspecies based on all against protein comparison to define homologs. A hit is considered significant if 50%/50% (identity/length coverage) requirement between-proteomes is met. Paralogs (internal homology) are proteins within a genome matching the same 50–50 rule. Cms, *C*. *michiganesis* subsp. *sepedonicus*; Cmn, *C*. *m*. subsp. *nebraskensis*; Cmm, *C*. *m*. subsp. *michiganensis*; Cmi, *C*. *m*. subsp. *insidiosus*; *C*. *m*. subsp. *capsici*.
(PPTX)

S1 Table Number of protein-coding sequences related to stress response survival capacity of the five subspecies of *Clavibacter michiganensis*. (PPTX)
