# Supplementary material for: Comparative genomics of Clavibacter michiganensis subspecies, pathogens of important agricultural crops
Source: PLoS One. 2017 Mar 20;12(3):e0172295. doi: 10.1371/journal.pone.0172295 (PMC5358740; doi:10.1371/journal.pone.0172295)
Supplement: S1 Fig — The optimal tree with the sum of branch length = 0.11186829 is shown. The values next to the branches are percentage of replicate trees in which the associated taxa clustered together in the bootstrap test (1000 replicates). Bootstrap values greater than 50% are shown. The tree is drawn to scale, with branch lengths in the same units as those of the evolutionary distances used to infer the phylogenetic tree. The evolutionary distances were computed using the Kimura 2-parameter method. The analysis involved 17 nucleotide sequences. All positions containing gaps and missing data were eliminated. There were a total of 1,250 positions in the final dataset. Taxa in bold are strains used in genome comparison that are not type strains and clustered perfectly with the corresponding type strains. The sequence accession numbers of the taxa are given in parentheses. (PPTX) [file pone.0172295.s006.pptx]

## Slide 1
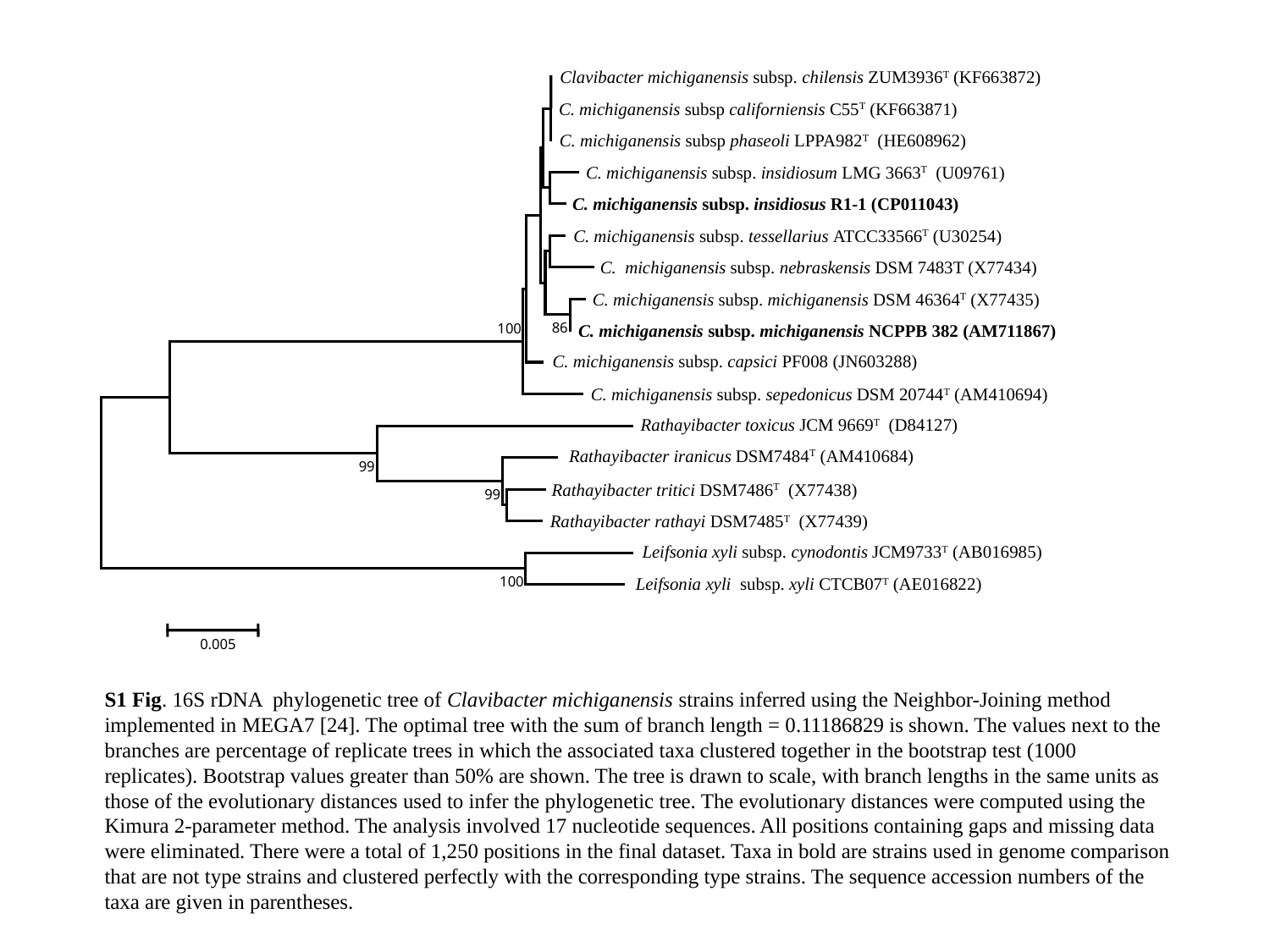

Clavibacter michiganensis subsp. chilensis ZUM3936T (KF663872)
 C. michiganensis subsp californiensis C55T (KF663871)
C. michiganensis subsp phaseoli LPPA982T (HE608962)
 C. michiganensis subsp. insidiosum LMG 3663T (U09761)
 C. michiganensis subsp. insidiosus R1-1 (CP011043)
 C. michiganensis subsp. tessellarius ATCC33566T (U30254)
C. michiganensis subsp. nebraskensis DSM 7483T (X77434)
 C. michiganensis subsp. michiganensis DSM 46364T (X77435)
86
100
 C. michiganensis subsp. michiganensis NCPPB 382 (AM711867)
C. michiganensis subsp. capsici PF008 (JN603288)
C. michiganensis subsp. sepedonicus DSM 20744T (AM410694)
Rathayibacter toxicus JCM 9669T (D84127)
Rathayibacter iranicus DSM7484T (AM410684)
99
Rathayibacter tritici DSM7486T (X77438)
99
Rathayibacter rathayi DSM7485T (X77439)
Leifsonia xyli subsp. cynodontis JCM9733T (AB016985)
 Leifsonia xyli subsp. xyli CTCB07T (AE016822)
100
0.005
S1 Fig. 16S rDNA phylogenetic tree of Clavibacter michiganensis strains inferred using the Neighbor-Joining method implemented in MEGA7 [24]. The optimal tree with the sum of branch length = 0.11186829 is shown. The values next to the branches are percentage of replicate trees in which the associated taxa clustered together in the bootstrap test (1000 replicates). Bootstrap values greater than 50% are shown. The tree is drawn to scale, with branch lengths in the same units as those of the evolutionary distances used to infer the phylogenetic tree. The evolutionary distances were computed using the Kimura 2-parameter method. The analysis involved 17 nucleotide sequences. All positions containing gaps and missing data were eliminated. There were a total of 1,250 positions in the final dataset. Taxa in bold are strains used in genome comparison that are not type strains and clustered perfectly with the corresponding type strains. The sequence accession numbers of the taxa are given in parentheses.
