# Supplementary material for: Comparative genomics of Clavibacter michiganensis subspecies, pathogens of important agricultural crops
Source: PLoS One. 2017 Mar 20;12(3):e0172295. doi: 10.1371/journal.pone.0172295 (PMC5358740; doi:10.1371/journal.pone.0172295)
Supplement: S2 Fig — Data of DNA, RNA and gene annotation are from the published GenBank entries. Each lane of the circular representation of the chromosome shows a different DNA feature. From innermost circle: size of genome (axis), percent AT (red = high AT), GC skew (blue = most G’s; orange arrows), inverted and direct repeats (color = repeats), position preference, stacking energy and intrinsic curvature. Dark brown arrows highlight areas of the genome with significantly different DNA structures than the remaining of the genome. Blue arrows shows the locations of rRNA operons as annotated in the GenBank file. Genome atlas was generated using CMG-Biotools [19] which calculates a numerical value for each nucleotide and saved in a file that is read by GeneWiz software. See “Materials and Methods” for details. (PPTX) [file pone.0172295.s007.pptx]

## Slide 1
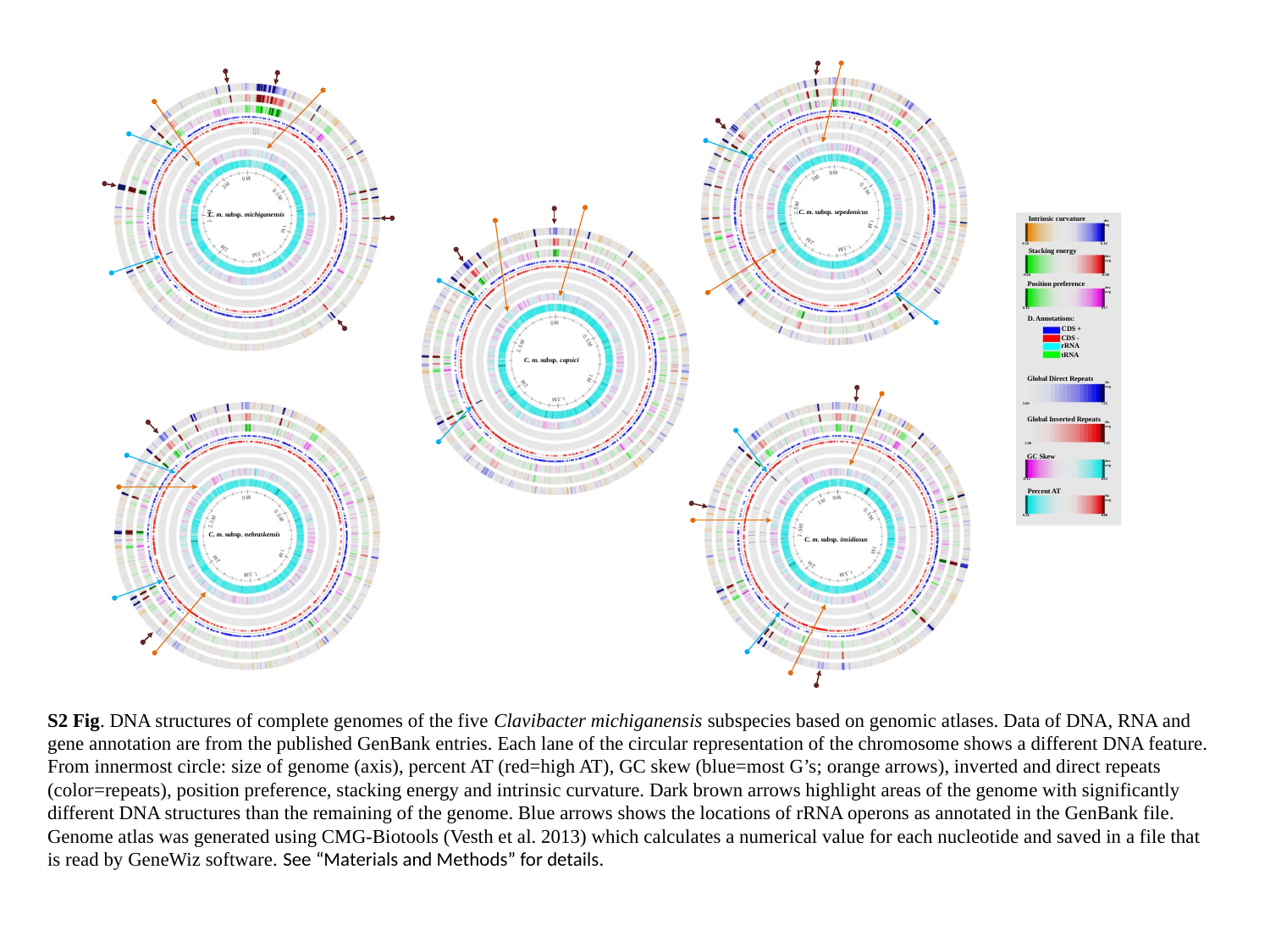

Intrinsic curvature
Stacking energy
Position preference
D. Annotations:
CDS +
CDS -
rRNA
tRNA
Global Direct Repeats
Global Inverted Repeats
GC Skew
Percent AT
dev
avg
0.10
0.14
-9.84
-8.98
0.14
0.17
5.00
7.25
5.00
7.25
-0.12
0.12
0.20
0.80
dev avg
dev avg
fix avg
fix avg
dev avg
fix avg
S2 Fig. DNA structures of complete genomes of the five Clavibacter michiganensis subspecies based on genomic atlases. Data of DNA, RNA and gene annotation are from the published GenBank entries. Each lane of the circular representation of the chromosome shows a different DNA feature. From innermost circle: size of genome (axis), percent AT (red=high AT), GC skew (blue=most G’s; orange arrows), inverted and direct repeats (color=repeats), position preference, stacking energy and intrinsic curvature. Dark brown arrows highlight areas of the genome with significantly different DNA structures than the remaining of the genome. Blue arrows shows the locations of rRNA operons as annotated in the GenBank file. Genome atlas was generated using CMG-Biotools (Vesth et al. 2013) which calculates a numerical value for each nucleotide and saved in a file that is read by GeneWiz software. See “Materials and Methods” for details.
