# Supplementary material for: Comparative genomics of Clavibacter michiganensis subspecies, pathogens of important agricultural crops
Source: PLoS One. 2017 Mar 20;12(3):e0172295. doi: 10.1371/journal.pone.0172295 (PMC5358740; doi:10.1371/journal.pone.0172295)
Supplement: S3 Fig — A hit is considered significant if 50%/50% (identity/length coverage) requirement between-proteomes is met. Paralogs (internal homology) are proteins within a genome matching the same 50–50 rule. Cms, C. michiganesis subsp. sepedonicus; Cmn, C. m. subsp. nebraskensis; Cmm, C. m. subsp. michiganensis; Cmi, C. m. subsp. insidiosus; C. m. subsp. capsici. (PPTX) [file pone.0172295.s008.pptx]

## Slide 1
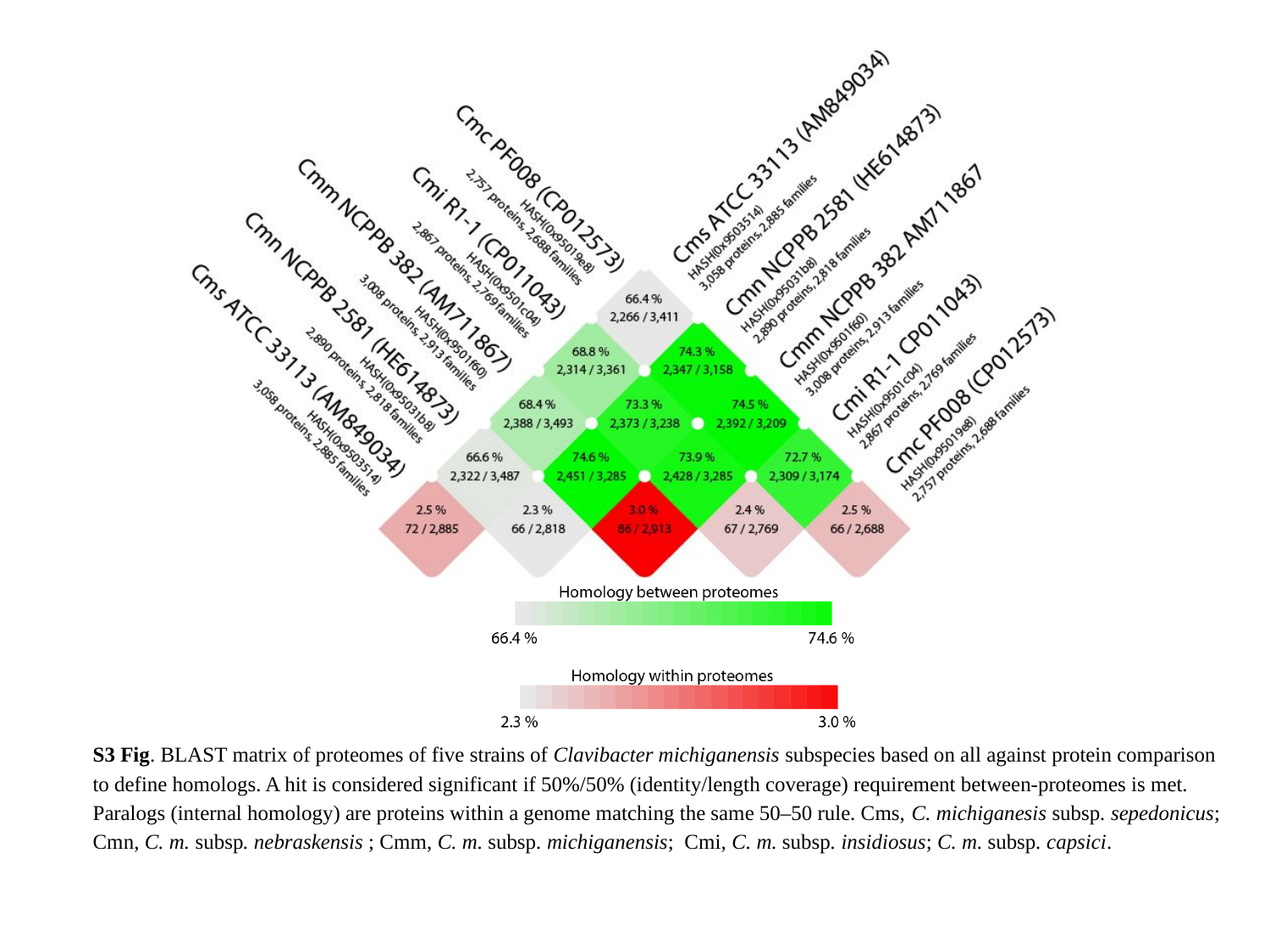

S3 Fig. BLAST matrix of proteomes of five strains of Clavibacter michiganensis subspecies based on all against protein comparison to define homologs. A hit is considered significant if 50%/50% (identity/length coverage) requirement between-proteomes is met. Paralogs (internal homology) are proteins within a genome matching the same 50–50 rule. Cms, C. michiganesis subsp. sepedonicus; Cmn, C. m. subsp. nebraskensis ; Cmm, C. m. subsp. michiganensis; Cmi, C. m. subsp. insidiosus; C. m. subsp. capsici.
