# Supplementary material for: Comparative genomics of Clavibacter michiganensis subspecies, pathogens of important agricultural crops
Source: PLoS One. 2017 Mar 20;12(3):e0172295. doi: 10.1371/journal.pone.0172295 (PMC5358740; doi:10.1371/journal.pone.0172295)
Supplement: S1 Table — (PPTX) [file pone.0172295.s009.pptx]

## Slide 1
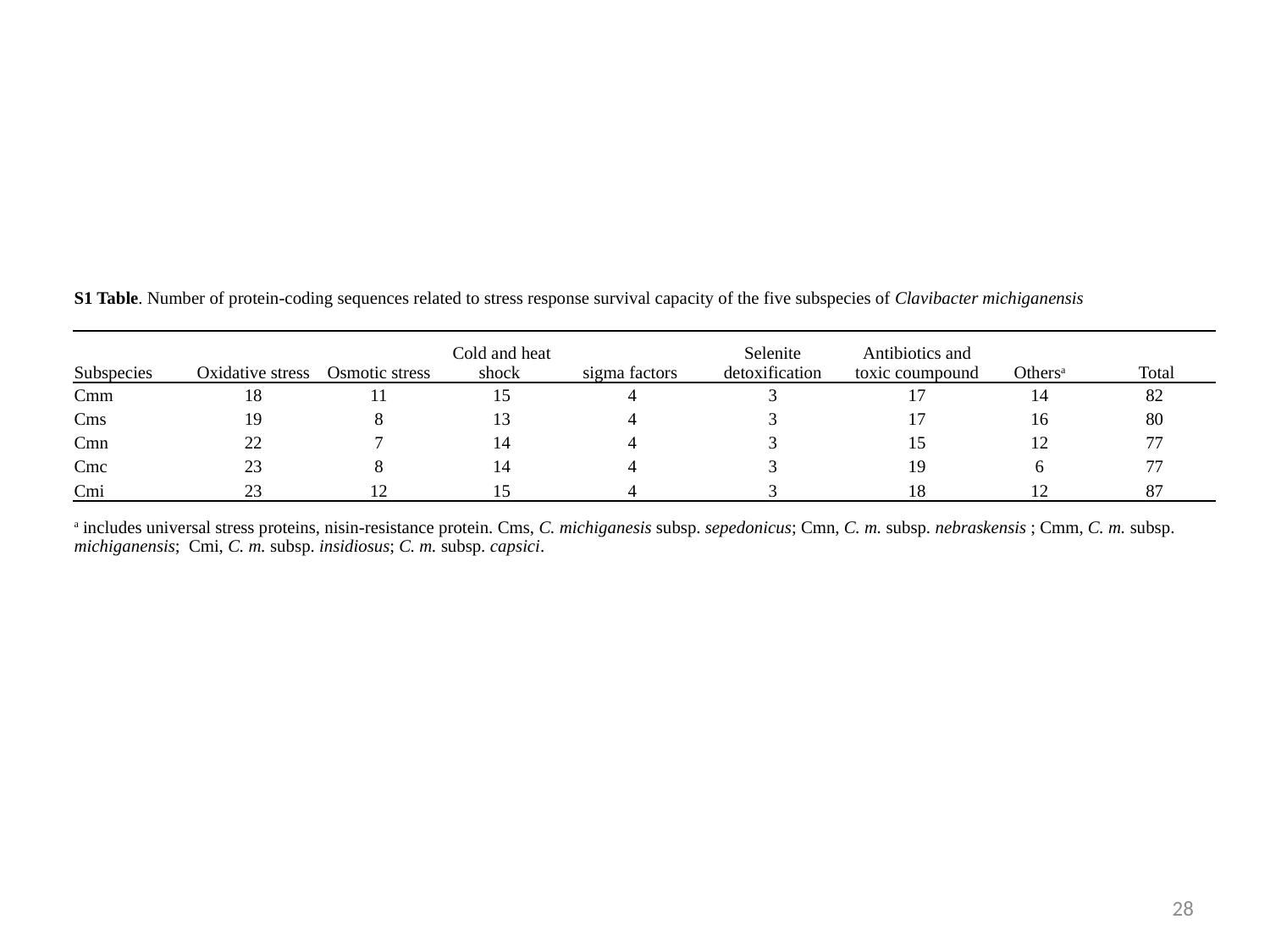

| S1 Table. Number of protein-coding sequences related to stress response survival capacity of the five subspecies of Clavibacter michiganensis | | | | | | | | |
| --- | --- | --- | --- | --- | --- | --- | --- | --- |
| | | | | | | | | |
| Subspecies | Oxidative stress | Osmotic stress | Cold and heat shock | sigma factors | Selenite detoxification | Antibiotics and toxic coumpound | Othersa | Total |
| Cmm | 18 | 11 | 15 | 4 | 3 | 17 | 14 | 82 |
| Cms | 19 | 8 | 13 | 4 | 3 | 17 | 16 | 80 |
| Cmn | 22 | 7 | 14 | 4 | 3 | 15 | 12 | 77 |
| Cmc | 23 | 8 | 14 | 4 | 3 | 19 | 6 | 77 |
| Cmi | 23 | 12 | 15 | 4 | 3 | 18 | 12 | 87 |
| a includes universal stress proteins, nisin-resistance protein. Cms, C. michiganesis subsp. sepedonicus; Cmn, C. m. subsp. nebraskensis ; Cmm, C. m. subsp. michiganensis; Cmi, C. m. subsp. insidiosus; C. m. subsp. capsici. | | | | | | | | |
28
